# Analyzing patient perspectives on orthodontic treatment through social media hashtags

**DOI:** 10.1371/journal.pone.0330263

**Published:** 2025-08-21

**Authors:** Letícia Machado Berretta, Gil Guilherme Gasparello, Evelise Machado de Souza, Giovani Ceron Hartmann, Ewandro Carlos Berretta, Elisa Souza Camargo, Sérgio Aparecido Ignácio, Orlando MotohiroTanaka

**Affiliations:** 1 Private Practice, Joinville, Santa Catarina, Brazil; 2 Orthodontics, Pontifícia Universidade Católica do Paraná, Medicine and Life Science School, Curitiba, Brazil; 3 Research Unit of Population Health, University of Oulu, Oulu, Finland; 4 Restorative Dentistry, Graduate Dentistry Program, Pontifícia Universidade Católica do Paraná, Medicine and Life Science School, Curitiba, Brazil; 5 Docent, Residency in Orthodontics, Pontifícia Universidade Católica do Paraná, Medicine and Life Science School, Curitiba, Brazil; 6 Biostatistics, Pontifícia Universidade Católica do Paraná, School of Medicine and Life Sciences, Curitiba, Brazil; 7 Orthodontics, Graduate Dentistry Program, Pontifícia Universidade Católica do Paraná, Medicine and Life Science School, Curitiba, Brazil; Universidade Federal do Rio de Janeiro, BRAZIL

## Abstract

Social media has become a key platform for health-related communication and patient experience sharing. In orthodontics, analyzing patient-generated content on social media helps uncover perceptions, motivations, and concerns related to treatment. Understanding the most commonly reported complaints allows orthodontists to offer solutions, enhancing overall patient satisfaction with treatment The objective of this study is to identify and categorize patient views on orthodontic treatment across social media, providing a broader understanding of global patients’ perspectives. The search was carried out on Instagram and X platforms using the hashtags #braces, #invisalign, #orthodontics, and #orthodontist. Data were collected over a seven-week period without restriction on languages or geographical locations. The qualitative analysis focused involved thematic categorization of posts content, while quantitative analysis used Pearson chi-square tests with Bonferroni-adjusted values, with a significance level of *p* < 0.05. A total of 18,605 posts were analyzed, with 96.60% (17,971 posts) from Instagram, and 3,4% (634 posts) from X platforms. Of the posts analyzed, 89.52% were made by dentists or dental clinics, 3.86% by dental product companies, and only 0.82% by patients undergoing orthodontic treatment. Five main themes were identified in patient posts: “positive accounts of orthodontic treatment” (40.53%), “enthusiasm about starting treatment” (20.26%), “reports of anxiety due to orthodontic treatment” (19.60%), “complaints and limitations about treatment” (16.34%), and “neutral accounts of orthodontic treatment” (3.27%). No statistically significant associations were found in the nature of content between the two platforms (*p* > 0.05). Dentists and clinics dominate social media discussions on orthodontics. Among patient-shared content, ‘positive experiences’ were the most frequently mentioned. No significant differences were observed between platforms in the type of posts analyzed.

## Introduction

Currently, social media has 4.88 billion active users worldwide, representing over 60% of the global population [1]. This widespread adoption can be attributed to the platforms’ facilitation of dynamic communication and real-time experience sharing among users [2]. Initially, online platforms primarily focused on one-way information dissemination via portals and websites but have since evolved into interactive spaces that cultivate interpersonal connections [3]. Thus, social media relies heavily on user collaboration in both content creation and post sharing [4].

Regarding healthcare, the widespread use of social media has enabled its application in the academic field as a valuable source of scientific research [5–7]. Specifically, in the field of orthodontics, studying patients’ behavior and their shared content on social media platforms can provide comprehension into their perspectives on treatments, motivations, and concerns [8]. Understanding the most commonly reported complaints allows orthodontists to offer solutions, enhancing overall patient satisfaction with treatment [9].

Furthermore, social media has become a pivotal tool for patients seeking information about dental treatments [10]. Inquiries about orthodontic procedures on these platforms often revolve around expected outcomes, types of appliances, and practical details concerning the treatment process [11]. Engagement on social media engagement relies on hashtags, which categorize content, enhance visibility, and connect users with relevant topics. Hashtags influence algorithms, expanding reach and boosting engagement, making them essential for understanding social media trends [11].

Social media engagement relies on hashtags, which categorize content, enhance visibility, and connect users with relevant topics. Hashtags influence algorithms, expanding reach and boosting engagement, making them essential for understanding social media trends [11].

Previous studies have observed that patient comments on social media become more positive as orthodontic treatment progresses towards completion [12]. For instance, a study analyzing Twitter posts related to Invisalign treatment found that pre-treatment posts were predominantly positive, highlighting patients’ expectations, whereas treatment-phase posts exhibited a balance of positive and negative sentiments, with negative posts often concerning pain, compliance issues, and impacts on diet and pronunciation (9). Another study on English-language social media posts found that, in clear aligner therapy, patients express high expectations before treatment, while more negative experiences emerge during treatment that are shared on social media [9]. Additionally, an analysis of German-language posts revealed that platform-specific factors influence user behavior [13].

Despite the existing evidence, comprehensive research on patient perspectives in orthodontic treatment across social media platforms, without geographical or language limitations, remains limited. Therefore, the objective of this study is to identify and categorize patient views on orthodontic treatment across social media, providing a broader understanding of global patients’ perspectives.

## Methods

This cross-sectional, observational study focused on the content assessment of orthodontics-related posts on the social media platforms Instagram and X (formerly known as Twitter). Since this research solely involves the examination of publicly accessible posts, it was determined that submission for approval by the Research Ethics Committee was deemed unnecessary.

### Data collection

In preparation for the main study, a pilot study was conducted on the platforms in January 2023. This preliminary investigation aimed to assess the feasibility of the research and identify the most frequently used hashtags related to orthodontics. The pilot phase guided the selection of hashtags, #braces, #invisalign, #orthodontics, and #orthodontist, and helped establish standardized procedures for data collection and post categorization. Furthermore, the pilot study enabled the researchers to calibrate their interpretation of posts, ensuring consistency and reliability during the main study.

The search strategy was consistent across both Instagram and X, using the predetermined hashtags: #braces, #invisalign, #orthodontics, and #orthodontist. These hashtags were chosen based on their relevance and frequency identified on the pilot study [5,12].

Data collection was carried out by two trained researchers, LMB and GGG, who had gained essential skills and insights during the pilot study. In cases of any disagreement between these researchers, a third researcher, OT, was consulted to reach a consensus [5,14,15].

To ensure a comprehensive and varied dataset, posts were systematically collected every day for a period of 7 weeks, from February 15th to April 5th, 2023. This method involved gathering data on a specific day of the week in each week – Mondays in the first week, Tuesdays in the second, and so forth. This sequential approach aimed to cover all seven days of the week by the study’s conclusion to ensure a diverse range of posts from different users and to represent each day in the analysis. This strategy maximized the breadth and diversity of the dataset [16].

Posts were included in the study based on specific criteria: they needed to contain content directly related to orthodontics. This inclusion criterion was applied without restrictions on language or geographical location, ensuring a broad and diverse dataset. The research team’s multilingual capabilities played a crucial role in this process. The researchers were fluent in English, Spanish, Portuguese, Italian, German, and French. For languages beyond their fluency the researchers used the Google Translate (available at https://translate.google.com) for accurate translation and interpretation of the text [17,18]. This multilingual approach was essential for a thorough and accurate analysis of the orthodontics-related content [5].

Regarding the exclusion criteria, posts were omitted if they were not relevant to dentistry. Additionally, ambiguous, meaningless or duplicated content was excluded. These measures helped maintain the study’s focus and ensure the quality and relevance of the data collected.

### Data analysis

The study utilized a mixed methods research approach, blending qualitative and quantitative methodologies to ensure a thorough and multifaceted analysis of the data. Initially, a structured qualitative content analysis was conducted on all collected posts [13,19].

Subsequently, the posts were classified into several distinct categories: positive experiences with orthodontic treatment, complaints and limitations regarding treatment, expressions of enthusiasm about beginning treatment, accounts of anxiety related to orthodontic procedures, and neutral, unbiased descriptions of orthodontic experiences.

### Statistical analysis

The collected data were tabulated in an electronic database using Microsoft Excel (Microsoft, Inc., Redmond, WA, USA). Statistical analysis was performed using SPSS version 25 (IBM, Armonk, USA). Quantitative analyses were employed to investigate potential differences between the content of posts on the X and Instagram platforms, as well as between the identified themes and the hashtags used in the posts. The Pearson chi-square independence test was utilized, Bonferroni adjustments were made to account for multiple comparisons across the variables of social media platforms, types of accounts, and hashtags. A significance level of 0.05 was considered.

## Results

**Fig 1:**
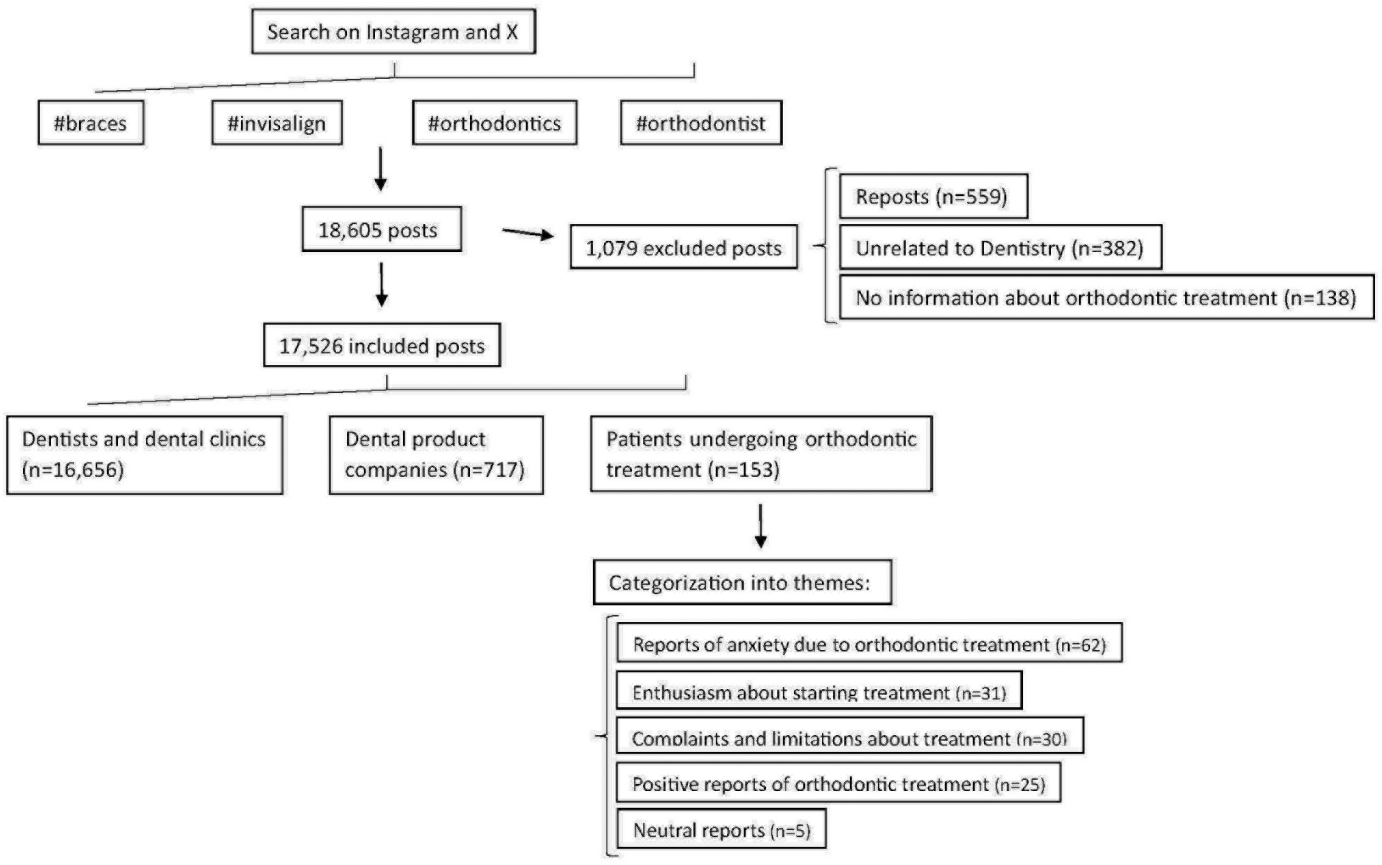
Flowchart with description of the data collected.

A total of 18,605 posts were identified on the X and Instagram platforms. Of these, a substantial 16,656 posts (89.52%) were published by dentist and dental clinic profiles, primarily for advertising and service promotion. Additionally, 717 (3.86%) were from dental product companies, 559 (3.00%) were reposts or duplicates, and 382 posts (2.06%) were unrelated to dentistry. Furthermore 138 (0.74%) did not express any opinion on orthodontic treatment, while 153 (0.82%) were from patients sharing their opinions about orthodontic treatment, as illustrated in Fig 1.

Patients undergoing orthodontic treatment were identified across ten different languages. English was the predominantly language (81.70%, n = 125), followed by Japanese (7.19%, n = 11), German (2.62%, n = 4), Chinese (1.96%, n = 3), Spanish (1.96%, n = 3), Malay (1.31%, n = 2), Portuguese (1.31%, n = 2), Danish (0.65%, n = 1), Italian (0.65%, n = 1), and Ukrainian (0.65%, n = 1).

**Fig 2:**
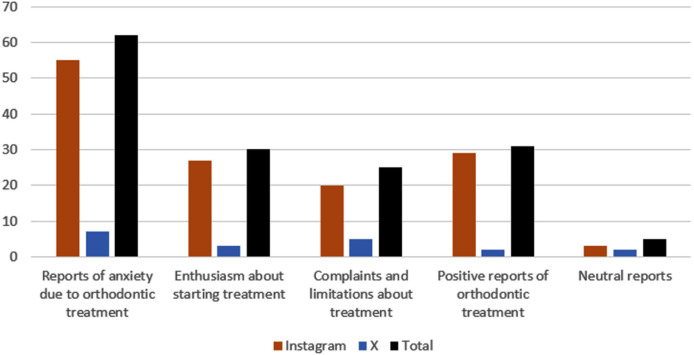
Thematic categorization of patient posts.

All 153 posts from patients undergoing orthodontic treatment were classified as follows: ‘positive accounts of orthodontic treatment’ (40.53%, n = 62), ‘enthusiasm about starting treatment’ (20.26%, n = 31), ‘reports of anxiety due to orthodontic treatment’ (19.60%, n = 30), ‘complaints and limitations about treatment’ (16.34%, n = 25), and ‘neutral accounts of orthodontic treatment’ (3.27%, n = 5) (Fig 2).

The theme ‘reports of anxiety due to orthodontic treatment’ included narratives expressing anxiety about initiating treatment, fear of pain, concerns about the outcome, and anxiety regarding the completion of treatment. In contrast, the theme ‘enthusiasm about starting treatment’ comprised narratives expressing excitement about commencing treatment or joy at the installation of orthodontic appliances.

The theme ‘complaints and limitations about treatment’ encompassed reports of pain, appliance breakage, embarrassment about the appliance, complaints about the treatment costs, and difficulties in speech and eating. On a more positive note, ‘positive reports of orthodontic treatment’ featured posts that expressed joy in choosing the color of the appliance, happiness with the progress of treatment, and satisfaction with the outcome or completion of treatment. Additionally, posts that discussed orthodontic treatment without specifically expressing a positive or negative sentiment were categorized under the ‘neutral reports’ theme. Examples of posts from each category are detailed in Table 1.

**Table 1:** Examples of posts according to each category.

Reports of anxiety due to orthodontic treatment	Post #65*: I can’t wait until I get my #braces.* Post #98: *A quick update: 7 trays left to go. Can’t wait!!*
Enthusiasm about starting treatment	Post #4: *Just got braces, yay #braces #lund #yay.* Post *#114: Super excited to have my braces on!! Things are going great so far! #braces #excited*
Complaints and limitations about treatment	Post #139: *Braces hurts so bad after my every appointment with dentist… #braces.* Post #144: *My insurance is covering about $2,000 of my #Invisalign, but I’m sitting here like “Ok, but who gon pay the other thousands??”. No international vaca for me this year...*
Positive reports of orthodontic treatment	Post #6: *Braces progress! About 2 months of braces and the large front gap is gone. #adultbraces #braces #dentaljourney.* Post #78: *I’m done with Invisaling Aligners! My teeth are now in their final positions. I’m super happy with the results! I can’t stop smilling. #invisaling #smile*
Neutral reports	Post #98: *Hi! A quick update. 7 trays left to go! #invisaling.* Post #146: Tray 19/60. *Almost reaching 1/3 of the treatment. Now, the duration I wear the trays decreases from 15 to 10 days since they move the teeth less. Let’s go. #invisalign.*

At the end of data collection period, a total of 17,971 posts (96.60%) were identified on Instagram, compared to 634 posts (3.40%) on platform X. Specifically on Instagram, 16,179 posts (86.96%) were from dentists and dental clinics, whereas on X, there were 477 such posts (2.56%). Additionally, Instagram, 134 posts (0.72%) from patients undergoing orthodontic treatment, in contrast to X, where only 19 posts (0.10%) were identified.

**Fig 3:**
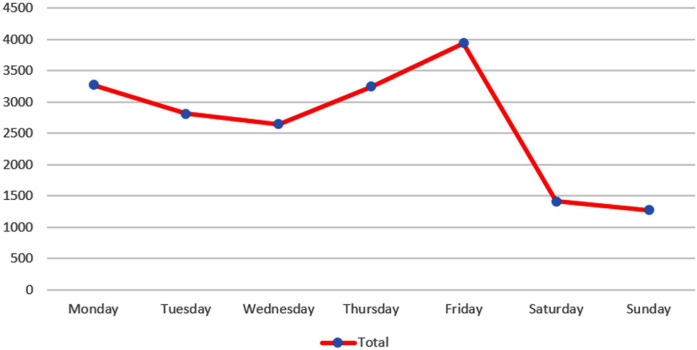
Data collected according to the days of the week.

**Fig 4:**
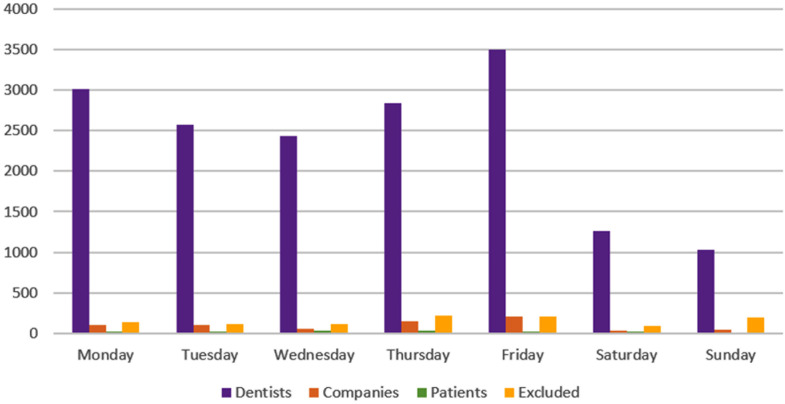
Volume of posts according to the days of the week.

The comparison between the volume of posts and the days of the week revealed that the day with the highest number of posts was Friday (21.12%, n = 3,940). Sunday had the lowest number of posts (6.85%, n = 1,275). Figs 3 and 4 describe the data collection according to each day of the week.

During the data collection, the hashtag #invisalign comprised the highest number of posts (n = 6,485), followed by #orthodontics (n = 5,683), #braces (n = 4,183), and lastly, #orthodontist (n = 2,254) in descending order. Among posts from dentists, #invisalign again led with 6,021 posts, followed by #orthodontics (n = 5,058), #braces (n = 3,687), and #orthodontist (n = 1,890). For posts from patients undergoing orthodontic treatment, the most frequent hashtag was #braces (n = 77), succeeded by #invisalign (n = 50), #orthodontics (n = 20), and #orthodontist (n = 6).

The revealed no significant differences in the distribution of post content between Instagram and X. Additionally, the quantity of posts within each identified theme did not vary significantly (p > 0.05), as detailed in Table 2. Furthermore, the analysis indicated no significant discrepancies between the investigated hashtags and the themes identified in the posts (p > 0.05), as presented in Table 3.

**Table 2:** Statistical analysis with cross-tabulation between the identified themes.

			Theme					
			Positive reports of orthodontic treatment	Complaints and limitations about treatment	Enthusiasm about starting treatment	Reports of anxiety due to orthodontic treatment	Neutral reports	Total
Social Media	Instagram	Count	55_a_	20_a_	29_a_	27_a_	3_a_	134
		% In Social Media	41%	14.9%	21.6%	20.1%	2.2%	100%
		% in Theme	88.7%	80%	93.5%	90%	60%	87.6%
	X	Count	7_a_	5_a_	2_a_	3_a_	2_a_	19
		% In Social Media	36.8%	26.3%	10.5%	15.8%	10.6%	100%
		% in Theme	11.3%	20%	6.5%	10%	40%	12.4%
Total		Count	62	25	31	30	5	153
		% In Social Media	40.5%	16.3%	20,.2%	19.6%	3.3%	100%
		% in Theme	100%	100%	100%	100%	100%	100%

Each subscript letter indicates a subset of Theme categories whose column proportions do not differ significantly from each other at the.05 level.

**Table 3:** Comparison between hashtags and identified themes.

			*Hashtag*				
			*Braces*	*Invisalign*	*Orthodontics*	*Orthodontist*	Total
Theme	Positive reports of orthodontic treatment	Counts	31_a_	23_a_	6_a_	2_a_	62
		% ein Theme	50%	37.1%	9.7%	3.2%	100%
		% in Hashtag	40.3%	46%	30%	33.3%	40.5%
	Complaints and limitations about treatment	Count	11_a_	11_a_	2_a_	1_a_	25
		% in Theme	44%	44%	8%	4%	100%
		% in Hashtag	14.3%	22%	10%	16.7%	16.3%
	Enthusiasm about starting treatment	Count	17_a_	6_a_	7_a_	1_a_	31
		% in Theme	54.8%	19.4%	22.6%	3.2%	100%
		% inHashtag	22.1%	12%	35%	16.7%	20.3%
	Reports of anxiety due to orthodontic treatment	Count	15_a_	8_a_	5_a_	2_a_	30
		% in Theme	50%	26.7%	16.7%	6.7%	100%
		% in Hashtag	19.5%	16%	25%	33.3%	19.6%
	Neutral reports	Count	3_a_	2_a_	0_a_	0_a_	5
		% in Theme	60%	40%	0%	0%	100%
		% in Hashtag	3.9%	4%	0%	0%	3.3%
Total		Count	77	50	20	6	153
		% in Theme	50.3%	32.7%	13.1%	3.0%	100%
		% in Hashtag	100%	100%	100%	100%	100%

Each subscript letter indicates a subset of Hashtag categories whose column proportions do not differ significantly from each other at the.05 level.

## Discussion

This study aimed to investigate social media posts related to orthodontics and identify perspectives on orthodontic treatment. The most common theme found in the study was ‘positive report about orthodontic treatment’ (40.53%), which includes happiness with the progress of the treatment, visualization of the results and completion of the treatment. This finding is in line with the literature which reports that, despite criticism and complaints throughout the treatment, 84.90% of adult patients finish orthodontic treatment with satisfaction [20].

Reports of anxiety and reports of complaints and limitations about orthodontic treatment comprised 35.94% of patient publications in the study. Often, patients tend to start treatment with high expectations and demands, especially regarding the technique with clear aligners [9]. It is, therefore, the orthodontist’s responsibility to provide clear and objective guidance on the risks and benefits of treatment, enabling patients to make informed decisions with realistic expectations [21].

These findings are consistent with recent literature emphasizing the dominance of commercially driven content in orthodontics-related posts on social media, especially on Instagram. The high proportion of posts created by dental professionals and clinics using hashtags for promotional purposes reflects a broader pattern reported in various healthcare fields, where professionals increasingly utilize social media platforms for marketing and branding [22,23]. In contrast, authentic patient-generated content remains limited and, in some cases, may be influenced by professional encouragement or incentives, potentially compromising the neutrality and credibility of these narratives [24]. Notably, there has been a marked increase in engagement by both patients and professionals on platforms such as X and Instagram, reflecting shifting user behavior and the growing relevance of these digital environments in healthcare communication [25]. These findings underscore the need for ethical and transparent communication strategies, as the blending of marketing with patient interaction can significantly influence public perception, trust, and treatment expectations.

No significant differences were identified between the content shared on social networks X and Instagram. This result may be explained due to the disproportionate number of posts on these platforms during the same timeframe, with Instagram comprising 96.60% of posts. The finding contrasts with one study that identified more positive reports on Instagram, significantly differing from platform X [13]. However, this previous study’s methodology was confined to analyzing only German-language posts.

Instagram and X were chosen for this study because both platforms offer searchable hashtags and generate high volumes of relevant data [6,10]. Unlike other social media platforms, Instagram and X are social networks with real-time publications. In this way, it is possible to observe patients’ perspectives with greater precision and reduced bias, based on reports of experiences at the moment they are experienced [16]. It is also noted that patients prefer to share their experiences on social media rather than communicate them to the orthodontist [8].

Among the 18,605 posts investigated, 89.52% were made by dentists or dental clinics and 93.38% had some commercial purpose, whether advertising for dentists or advertising for the sale of dental products. These numbers are greater than the presence of lay patients on the same hashtags and platforms investigated. It has been found that posts for dental advertising purposes have little or no control over the quality of the content published [5].

Many studies have focused on analyzing the quality of health-related information available online, including on search engines and social media platforms [26,27] Alarmingly, orthodontists themselves have sometimes been unable to distinguish between scientifically supported and unsupported posts [28]. The overall quality of orthodontic information on social media has been considered poor. Due to the lack of regulation or oversight, content may fail to reflect the best available evidence and should therefore be interpreted with caution. The content found may not provide the best scientific evidence available [29], Given the large volume of posts for commercial purposes and as there is little control over the quality of this information, their contents should be interpreted with caution [30].

The proliferation of health-related content on social media platforms has introduced significant challenges concerning the dissemination of misinformation and ethical considerations in patient engagement. A substantial portion of orthodontic-related posts are generated by dental professionals or clinics, often serving promotional objectives. This blurring of lines between genuine patient experiences and marketing strategies raises ethical questions, particularly when patients are encouraged to share content that may be influenced by professional interests [24] Such practices can compromise the authenticity of patient narratives and potentially mislead the public regarding treatment expectations. Healthcare professionals must navigate these dynamics carefully, ensuring that promotional activities do not overshadow the imperative for accurate and ethical patient communication. As highlighted in recent studies, the ethical use of social media in healthcare necessitates a balance between professional promotion and the maintenance of trust and transparency with patients and the broader public [22,23]. A 2014 study revealed that only 1.5% of social media users sought information about orthodontic treatment on X [12].

However, recent evidence shows that both X and Instagram have become key platforms for the exchange of orthodontic information between patients and professionals [25,28] marking a significant shift in user behavior over the past decade. Instagram, followed closely by X, is now widely used by patients, professionals, and dental students to search for healthcare services and information [22,25]. Moreover, a comparative study found that orthodontists and dental students tend to prefer posts highlighting technical aspects, while laypeople respond more to aesthetic or emotional elements [25]. These findings suggest that the type of content shared can influence the credibility of professionals and the public’s willingness to seek treatment.

Finally, it is necessary to be aware of the risks associated with social media. Misinformation, or fake news, is one of the main problems, where the platforms dynamics results in rapid spread of false and poor quality content [31]. Another significant concern is in relation to the platforms’ algorithm, as it is not yet capable to differentiate between benign and harmful content, and can encourage the development of obsessive and compulsive behaviors [32]. Therefore, it is essential, therefore, that the use of social networks is carried out with caution and critical judgment.

The main limitation of this study was the use of English-only hashtags in the search strategy. Although no language restrictions were imposed, this choice resulted in the predominance of English-language posts, limiting the potential for intercultural or geographic comparisons. Another limitation was the exclusive focus on two social media platforms, Instagram and X, which may not fully represent the broader population of social media users or capture trends on emerging or regionally popular platforms. Additionally, due to the high volume of posts collected, it was not feasible to perform a detailed analysis of content published by orthodontists, nor to explore their specific communication strategies or professional viewpoints.

It is also important to acknowledge the potential for bias in patient-generated content, particularly in cases where posts may have been encouraged by dental clinics for promotional purposes. Such influence could compromise the authenticity and spontaneity of patient narratives. Furthermore, although this study primarily focused on thematic content, it did not include the analysis of engagement metrics such as likes, comments, and shares. This omission was due to technical limitations and the variability of algorithmic visibility across platforms, which could have introduced inconsistencies. Nevertheless, we recognize that engagement indicators may offer valuable insights into the reach, reception, and influence of orthodontic content. Finally, while the study did not aim to evaluate misinformation or ethically inappropriate guidance, we acknowledge their relevance to patient safety and professional integrity. Given the large volume of promotional posts identified, the potential for misleading or biased content warrants attention. Future studies should incorporate robust tools to assess the accuracy and ethical quality of health-related information shared on social media.

## Conclusion

This study demonstrated that the vast majority of social media posts related to orthodontics are created by dentists and dental clinics, predominantly for commercial purposes. Authentic patient-generated content remains scarce. Among patient posts, positive perceptions of orthodontic treatment were the most frequently expressed, followed by enthusiasm at treatment onset, reports of anxiety, and treatment-related complaints. The low representation of spontaneous patient narratives, coupled with the prevalence of promotional material, emphasizes the need for critical appraisal of online health content and ethical engagement strategies within the digital dental landscape.
